# Atypical Pediatric Presentation of Pilomatricoma

**DOI:** 10.7759/cureus.39909

**Published:** 2023-06-03

**Authors:** Cassandra Mohr, Jaime Tschen

**Affiliations:** 1 Dermatology, McGovern Medical School, Houston, USA; 2 Dermatology, St. Joseph Dermatopathology, Houston, USA

**Keywords:** pediatric tumor, head and neck tumors, diagnosis delay, scrofuloderma, pilomatricoma

## Abstract

Pilomatricomas are uncommon, benign tumors of the hair follicle, which are often misdiagnosed upon initial inspection. Here we describe the case of a 4-year-old boy who presented with a persistent draining tumor on the left side of his neck for approximately two years. The tumor was originally misdiagnosed as scrofuloderma but, eventually, our patient’s pilomatricoma was identified with biopsy and successfully treated with elliptical excision. We discuss the importance of considering pilomatricoma in the differential diagnosis.

## Introduction

Pilomatricomas are frequently misdiagnosed, benign tumors that derive from the hair follicle [1]. They most commonly present on the head and neck as a firm, solitary, slow-growing tumor with a normal to pearly white dermis [2]. The overlying skin may have a blue or red discoloration. A perforating pilomatricoma is a rare clinical variant, presenting as an ulcer or horn-like, crusted nodule [3-5]. Pilomatricomas are also often referred to as calcifying epitheliomas of Malherbe due to frequent calcification of the tumor [6]. On histology, perforating pilomatricomas show transepidermal elimination of the tumor [7].

Though the exact pathophysiology remains unknown, a genetic link has been described in previous studies [8,9]. A mutation in exon 3 of the B-catenin gene involved in hair follicle differentiation has been proposed as a possible mechanism. Additionally, it has been established that the prevalence of pilomatricomas in patients with myotonic dystrophy is higher than in the general population [10-12]. Patients with myotonic dystrophy are also more likely to have multiple pilomatricomas and positive family history. Classic pilomatricomas have a bimodal distribution, presenting typically in children or the elderly. Perforating pilomatricomas, however, are much more common in the elderly [13]. Pilomatricomas typically present in Caucasians and have a slightly higher prevalence among females. When treatment is indicated, surgical excision is the first-line method and commonly results in complete resolution of the lesion. In the approximately 5% of cases that do recur, incomplete resection is the usual culprit.

## Case presentation

A 4-year-old boy presented with a draining tumor on the left side of his neck, located posteriorly and retroauricularly. The tumor had persisted for the past two years. Several months prior to presentation, the lesion had ruptured the surface and was diagnosed as cutaneous tuberculosis (scrofuloderma). The patient took rifampin and doxycycline as prescribed for several months without any noticeable improvement.

**Figure 1:**
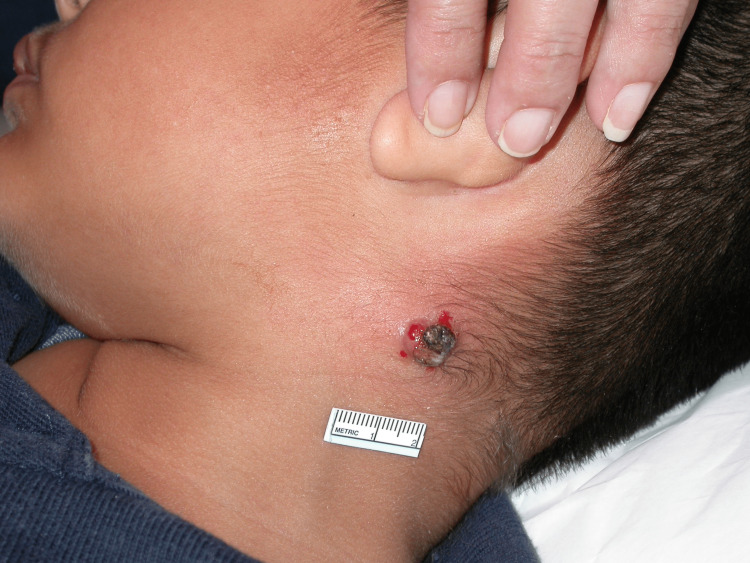
Ulcerated tumor in the patient's neck (distant view)

**Figure 2:**
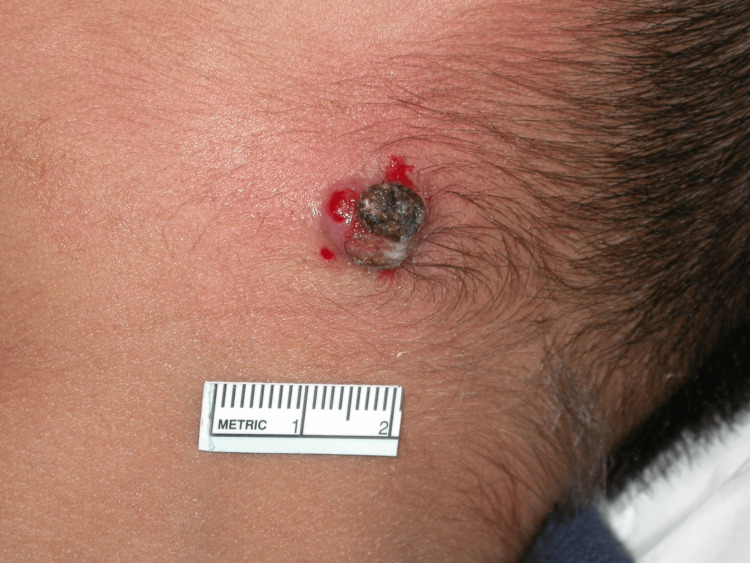
Ulcerated tumor in the patient's neck (closer view)

On physical exam, a 1.3 cm crusted draining tumor was appreciated on the left posterior neck (Figures 1-2). 

**Figure 3:**
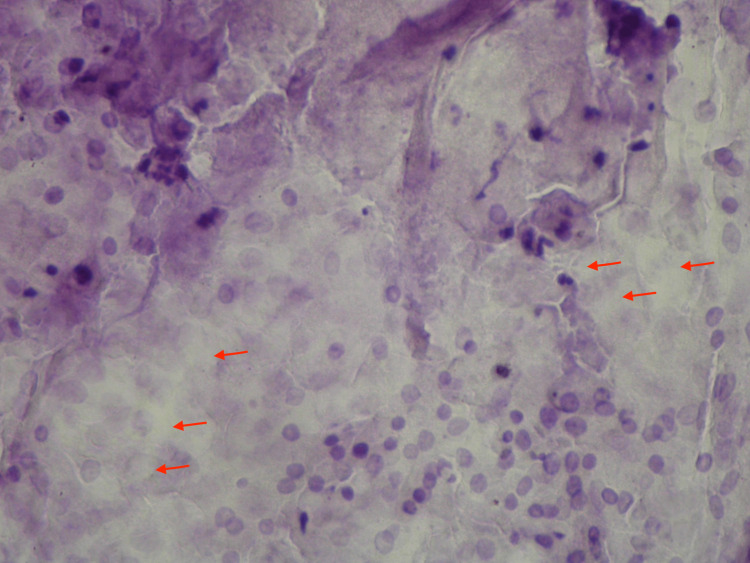
Smear of chalky material obtained after infiltration of anesthesia; characteristic ghost cells (arrows); hematoxylin and eosin 200x

Upon injection of the anesthetic for excisional biopsy, a white chalky substance was drained. A smear of the material performed prior to the biopsy showed epithelial cells with a few ghost cells (Figure 3).

**Figure 4:**
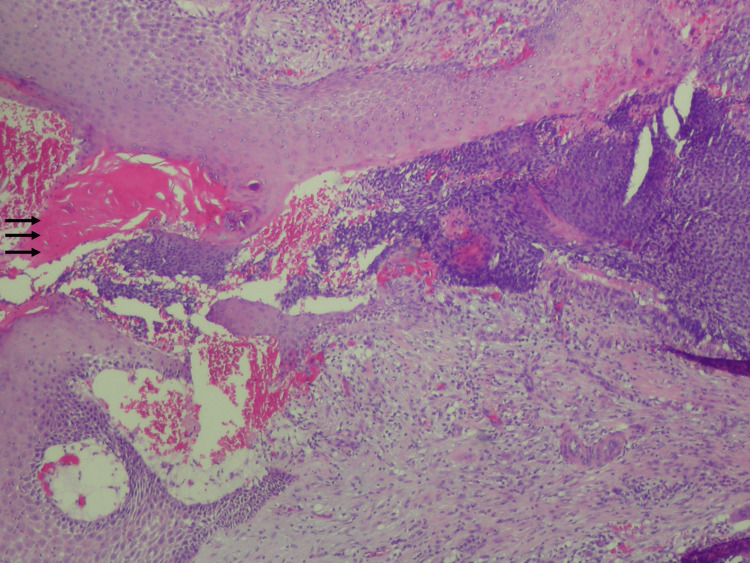
Perforating tumor (arrows) with disrupted epidermis; hematoxylin and eosin 10x Orientation: top of image is on the left.

**Figure 5:**
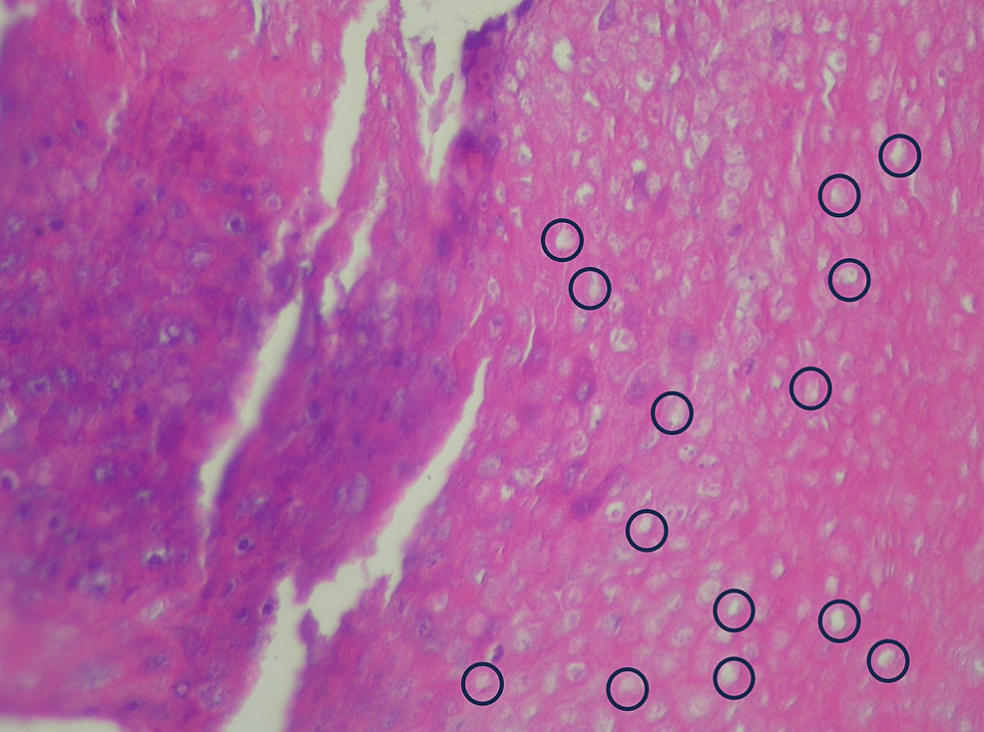
Characteristic ghost cells (circled) in tumor nest; hematoxylin and eosin 40x

The elliptical excision was completed without any complications. Final histopathology report of the epidermal channel described epithelial and ghost cells characteristic of a perforating pilomatricoma (Figures 4-5). A sinus containing tumor fragments with hemorrhage and both acute and chronic inflammation was seen. Moderate peripheral acanthosis was visualized at the opening of the sinus. The tumor showed no cytologic atypicalities and a small nasal oír (matricial) component, with the bulk of the tumor composed of sheets of ghost cells.

On follow-up at three months, the surgical site had healed without any evidence of recurrence.

## Discussion

Pilomatricoma was first described in 1880 by Malherbe and Chenantais [14]. It was originally suspected to originate from the sebaceous glands [15]. Over time, it was discovered that these benign tumors originated from the hair follicle, and the term 'pilomatricoma' came into use through Jones and Campbell in 1969 [16]. These cutaneous, firm lesions are often mistaken for similar yet more common lesions such as keratoacanthomas, neurofibromas, chondromas, and fibroxanthomas [1].

Our case of a rare clinical variant, perforating pilomatricoma, was incorrectly identified as scrofuloderma. This was likely due to the unique clinical characteristics of the perforating pilomatricoma involving tumor drainage and ulceration. The clinical differential diagnosis for a perforating pilomatricoma includes pyogenic granuloma, arteriovenous malformation, epidermal inclusion cyst, and soft tissue sarcomas [17].

Scrofuloderma, like pilomatricoma, often presents as a painless, subcutaneous, slow-growing lesion [18]. Scrofulodermas also typically present with purulent discharge which was consistent with our patient’s presentation. Scrofuloderma is diagnosed with a skin biopsy, showing multinucleated giant cells, caseous necrosis, and mixed inflammatory cells. In our case, histopathology was consistent with a perforating pilomatricoma, presenting with islands of epithelial cells, ghost cells, and basophilic cells [19].

Our case demonstrates the importance of clinicopathological correlations. Lesions exhibiting ghost cells include pilomatricomas, craniopharyngiomas, calcifying cystic odontogenic tumors, and odontomas. However, the tumor location would only be characteristic of a pilomatricoma or pilomatrix carcinoma. Further, the head and neck location is characteristic of many benign and malignant tumors. However, the ghost cells and draining sinus narrowed the diagnosis to perforating pilomatricoma. 

The most reliable diagnostic modality for pilomatricomas remains biopsy with pathologic evaluation. Once diagnosed, pilomatricomas have an excellent prognosis following complete excision. No other treatment modalities provide a definitive diagnosis and cure. The recurrence rate after excision has been reported to be approximately 2.6% [20]. Correct diagnosis and early intervention of pilomatricomas are important to avoid potential transformation into a pilomatrix carcinoma. This transformation, however, is much more likely to occur on the head and neck of middle age to elderly patients and occurs very rarely in children. Nevertheless, the rate of malignant transformation of pilomatricomas is difficult to establish due to the difficulty in distinguishing the malignant transformation of a benign pilomatricoma from a de novo pilomatrix carcinoma.

## Conclusions

Pilomatricomas are rare, benign lesions that typically present on the head and neck region of children or the elderly. Our case was a rare perforating type which, likely due to the tumor’s ulcerating characteristics and the fact that our patient was not in the typical age category for this lesion, was improperly diagnosed as scrofuloderma.

A pilomatricoma should be in the differential diagnosis when presented with a firm, solitary, slow-growing tumor in the head and neck of a child or elderly adult. Ulceration and drainage of the lesion can be indicative of the rare perforating clinical variant even among the pediatric population. To ensure diagnostic accuracy and proper management of these lesions, it is best to perform a biopsy for pathological evaluation. Once diagnosed, pilomatricomas often resolve completely with surgical excision, as described in this case.
